# Image synthesis of interictal SPECT from MRI and PET using machine learning

**DOI:** 10.3389/fneur.2024.1383773

**Published:** 2024-06-25

**Authors:** Azin Shokraei Fard, David C. Reutens, Stuart C. Ramsay, Steven J. Goodman, Soumen Ghosh, Viktor Vegh

**Affiliations:** ^1^Centre for Advanced Imaging, University of Queensland, Brisbane, QLD, Australia; ^2^Royal Brisbane and Women’s Hospital, Brisbane, QLD, Australia; ^3^ARC Training Centre for Innovation in Biomedical Imaging Technology, Brisbane, QLD, Australia

**Keywords:** interictal SPECT, pre-surgical evaluations of epilepsy, cross modality image estimation, generative adversarial networks, deep learning, image synthesis

## Abstract

**Background:**

Cross-modality image estimation can be performed using generative adversarial networks (GANs). To date, SPECT image estimation from another medical imaging modality using this technique has not been considered. We evaluate the estimation of SPECT from MRI and PET, and additionally assess the necessity for cross-modality image registration for GAN training.

**Methods:**

We estimated interictal SPECT from PET and MRI as a single-channel input, and as a multi-channel input to the GAN. We collected data from 48 individuals with epilepsy and converted them to 3D isotropic images for consistence across the modalities. Training and testing data were prepared in native and template spaces. The Pix2pix framework within the GAN network was adopted. We evaluated the addition of the structural similarity index metric to the loss function in the GAN implementation. Root-mean-square error, structural similarity index, and peak signal-to-noise ratio were used to assess how well SPECT images were able to be synthesised.

**Results:**

High quality SPECT images could be synthesised in each case. On average, the use of native space images resulted in a 5.4% percentage improvement in SSIM than the use of images registered to template space. The addition of structural similarity index metric to the GAN loss function did not result in improved synthetic SPECT images. Using PET in either the single channel or dual channel implementation led to the best results, however MRI could produce SPECT images close in quality.

**Conclusion:**

Synthesis of SPECT from MRI or PET can potentially reduce the number of scans needed for epilepsy patient evaluation and reduce patient exposure to radiation.

## Introduction

Localisation of the seizure focus is a key aspect of the pre-surgical workup of individuals with drug-refractory epilepsy and routinely requires Magnetic Resonance Imaging (MRI), Positron Emission Tomography (PET), and Single Photon Emission Computed Tomography (SPECT) scans. Comparison of SPECT scans performed after the injection of a cerebral blood flow tracer during a seizure (ictal scans) with SPECT scans of tracer distribution during normal brain activity (interictal scans) reveal hyperperfusion in brain structures involved in seizure activity. The computation of the difference image between the ictal and interictal SPECT co-registered to MRI (SISCOM) is a routine approach for localising the seizure onset zone with a high level of sensitivity and specificity (1). The ictal SPECT scan is usually obtained during inpatient continuous video-EEG monitoring, when medication reduction or cessation is undertaken to provoke their habitual seizures. Because of the unpredictability of obtaining an ictal scan, one common workflow is to obtain the interictal SPECT scan only after a successful ictal scan has been acquired and because the interictal state is assumed to pertain following a period of 24 h seizure freedom, the interictal scan is commonly performed after video-EEG monitoring has been discontinued and the patient discharged from hospital. This leads to uncertainty around the representativeness of the scan of normal brain activity, especially in individuals with subclinical or unobserved seizures. The interictal SPECT scan also entails an additional radiotracer dose. Perissinotti et al. directly replaced the interictal SPECT scan with the individual’s PET scan (2) and found only 65% concordance in localisation of the seizure focus compared to when the interictal SPECT scan was used. We therefore sought to develop methods of generating interictal SPECT scans from already-acquired interictal PET and MRI images.

While cross-modality image synthesis using machine learning is a well-established area of research (3), it has not been investigated in the context of SPECT. To date, machine learning implementations have involved the synthesis of PET from MRI or CT, and using similar frameworks, CT was estimated from both MRI and PET. Image synthesis experiments using machine learning were founded on Convolutional Neural Networks (CNNs) (4–6). Generative Adversarial Networks (GANs) were invented later (7), and thus applied after CNNs were investigated (8–12). The use of GANs instead of CNNs results in estimated images of higher quality (3).

In image synthesis, a CNN is used to establish the intensity projection between two imaging modalities. Here, autoencoder networks are typically responsible for projecting image intensities and forming the new image (i.e., the generator). GANs are essentially CNNs with an adversarial component, wherein the generator network competes with a second network (i.e., the discriminator). The discriminator network is trained to distinguish between real and fake images. Through the adversarial loss function, the discriminator network forces the generator network to create more realistic images during training. Quantitative measures, namely Root Mean Square Error (RMSE), Structural Similarity Index Measure (SSIM), and Peak Signal-to-Noise Ratio (PSNR), standard image quality metrics, were used to compare implementations. Our extensive review of over 80 cross-modality image synthesis studies concluded that pix2pix is a reliable choice for synthesising cross-modality images, and performs as well as a cycle GAN for brain images (3). Irrespective of the medical imaging modalities synthesised, pix2pix performed in a consistent manner. Furthermore, SPECT image synthesis has not been performed to date. Hence, our work is limited to the use of pix2pix for synthesising interictal SPECT images from PET, MRI, and both PET and MRI images.

Irrespective of whether CNNs or GANs are used, datasets of sufficient size are required to achieve an adequate level of network training. Preparation of the training dataset involves transforming input and output images to a common space. Existing studies have used either native (11, 13–17) or template space (10, 18–20), but to our knowledge, the impact of choosing one space over another has not been evaluated to date. As such, it remains unclear whether registration to a template space prior to training a GAN improves image synthesis.

Our main contributions of this article are as follows:

- To date, SPECT brain images have not been synthesised from other imaging modalities;
- We have used a state-of-the-art approach to translate PET and MRI scans to synthesised SPECT images;
- This provides a method of generating interictal SPECT scans without actual scanning being performed, as a means of streamlining the management of epilepsy patients undergoing pre-surgical evaluation; and
- We establish the impact of choosing template space or native space for input images into the image synthesis machine learning framework.

## Materials and methods

### Data collection

Data collection commenced following Royal Brisbane & Women’s Hospital Human Research Ethics Committee (Brisbane, Australia) approval. Through the epilepsy clinic at the Royal Brisbane & Women’s Hospital, we identified 86 subjects diagnosed with refractory focal epilepsy within the last 20 years. Forty-eight subjects had standard PET, SPECT and MPRAGE T1-Weighted MRI images available. PET and SPECT scans were collected in the axial orientation, whereas T1 MRI were acquired in the sagittal orientation. We should note that the resolution and quality of PET and SPECT images changed over time, and Table 1 summarises the dimension and resolution variations across each imaging modality within the dataset.

**Table 1:** Summary of the dimension and resolution of the subject dataset collected.

SPECT		PET		MRI	
Dimension	Resolution (mm)	Dimension	Resolution (mm)	Dimension	Resolution (mm)
128 × 128 × 83	2.34 × 2.34 × 2.34	440 × 440 × 132	0.82 × 0.82 × 2.0	256 × 256 × 192	0.93 × 0.93 × 0.9
128 × 128 × 47	3.89 × 3.89 × 3.89	512 × 512 × 111	0.79 × 0.79 × 2.0	512 × 512 × 192	0.44 × 0.44 × 0.89
128 × 128 × 44	4.79 × 4.79 × 4.79	-	-	512 × 512 × 155	0.44 × 0.44 × 1.0
-	-	-	-	256 × 256 × 144	0.89 × 0.89 × 0.99

### Image pre-processing

**Figure 1:**
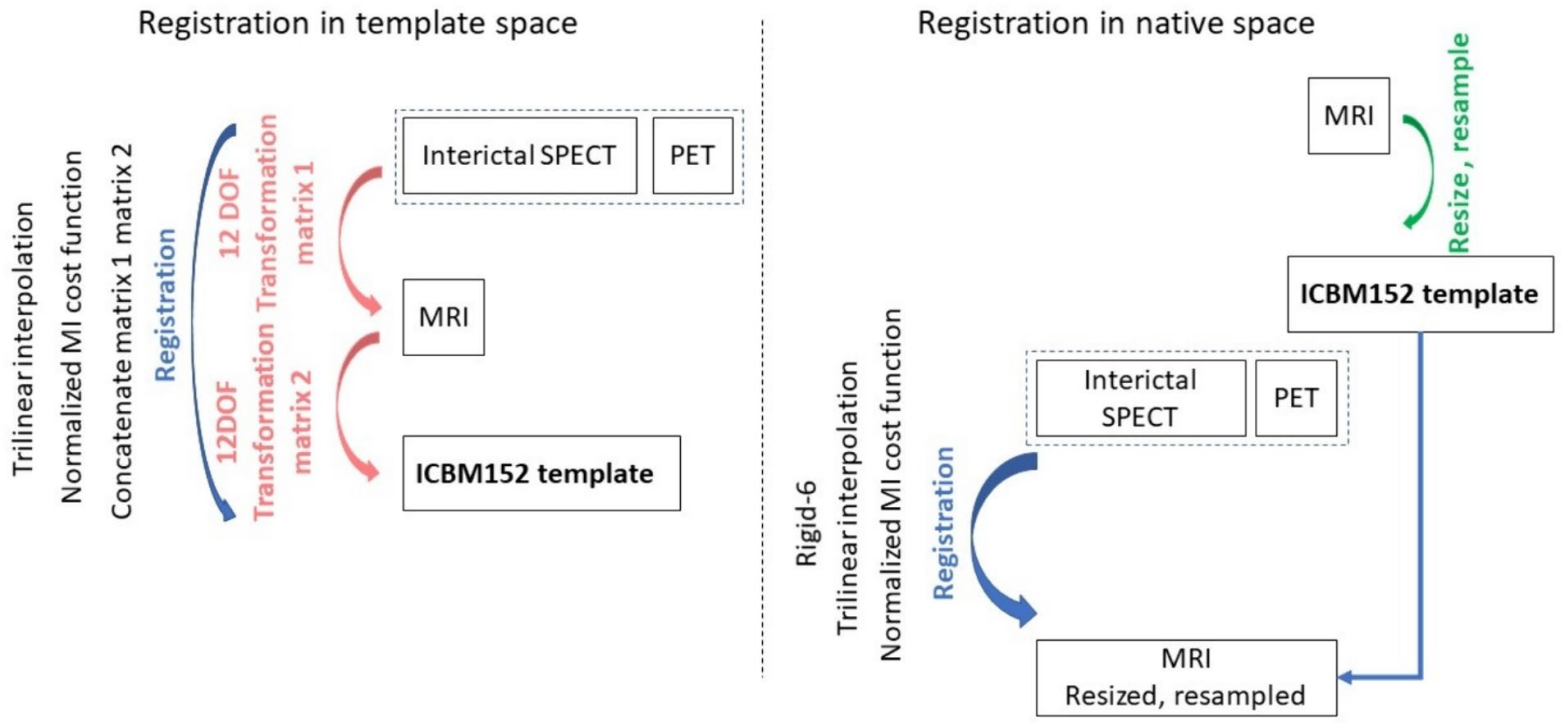
The medical image pre-processing steps used to prepare the machine learning input images. On the left the steps are shown for the case when images were registered to the template space, and on the right when images were aligned to the native space of the MRI acquisition.

All image processing methods were implemented using MIPAV (v10.0.0), SPM 12, and FSL 6.0.5.1. Using MIPAV, MRI images were reoriented to axial slices to match the PET and SPECT orientation. Individual brains were extracted from MRI and PET scans using the BET tool in FSL. The brains were extracted from the SPECT images using an intensity threshold based on the histogram computed in MIPAV. For experiments performed in native space (i.e., involving alignment of PET and SPECT images to MRI images in native space), all MRI images were resized to a 256 × 256 × 192 matrix and resampled to a resolution of 1 × 1 × 1 mm^3^ (3rd order Bspline in MIPAV). For each subject, PET and SPECT image volumes were aligned to the native space of the MRI image volume using rigid body registration in MIPAV, then resampled to the resolution of the MRI images. For experiments performed in template space, PET, SPECT, and MRI images were registered to the MNI ICBM152 space (21) using affine registration in MIPAV (automatic registration with 12 parameters), achieving a final matrix size of 256 × 256 × 192 and resolution of 1 × 1 x 1 mm^3^. Figure 1 depicts the steps involved in pre-processing the template and native space images prior to image synthesis. Irrespective of the experiment type, all images were standardised in a slice-by-slice manner to have their image intensities in the (−1, 1) range.

### GAN implementation

**Figure 2:**
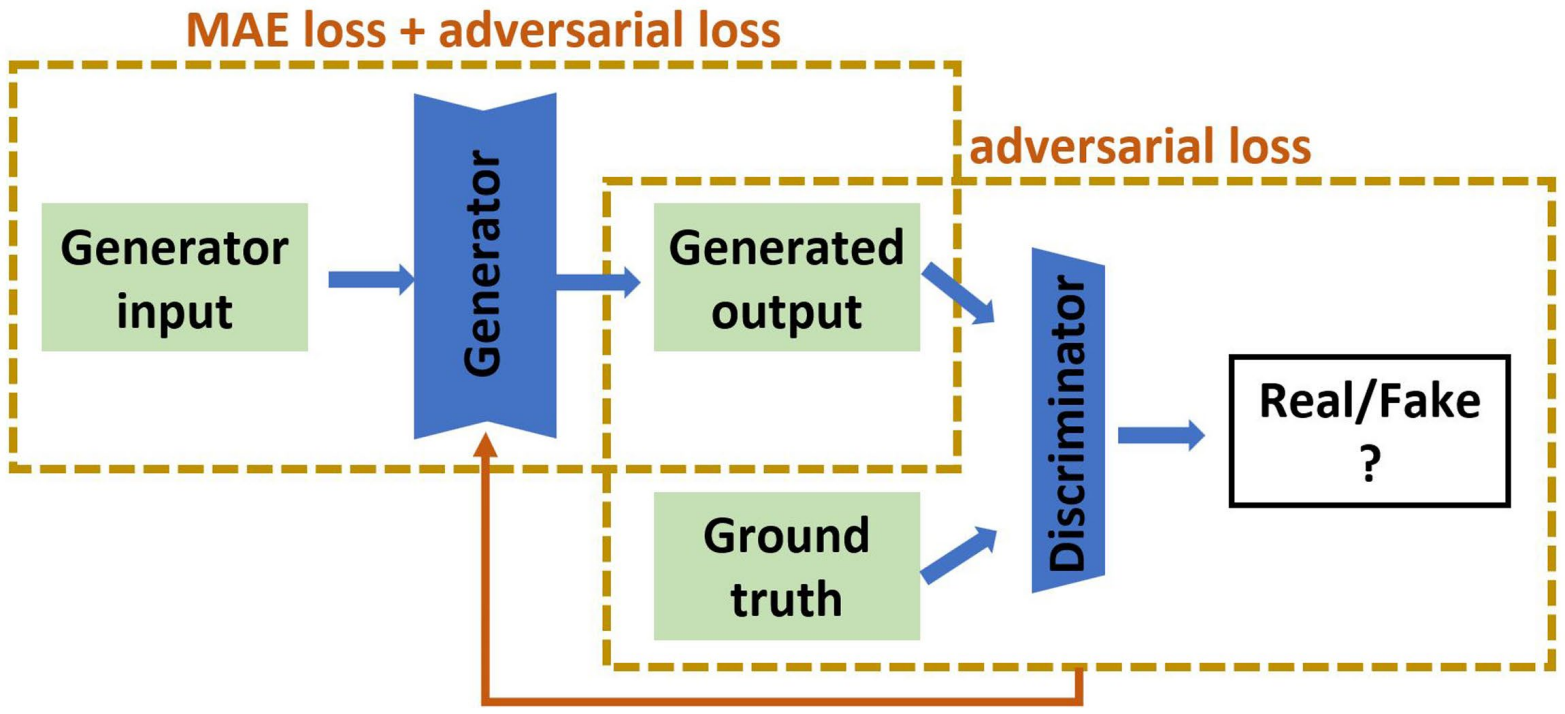
The pix2pix model used in this study. The input is of the form of MRI, PET or MRI and PET together, and the output is the synthetic SPECT image. The green parts are 2D medical images, the blue are artificial neural networks. MAE is mean absolute error.

Our recent review of cross-modality image estimation methods (3) concluded that GANs using the Pix2pix framework (22) are well suited to this task. Pix2pix is a supervised conditional GAN, which requires paired sets of input and target images for training (see Figure 2 for network structure). We implemented this framework to synthesise brain images under three distinct scenarios: (i) MRI to SPECT, (ii) PET to SPECT, and (iii) both MRI and PET to SPECT. The original conditional GAN described by Isola et al. (22) was implemented, and modified by changing the batch size to 8, and decreasing the learning rate from 0.001 to 0.0001.

Loss functions define how trainable weights evolve during training in a machine learning framework. Different GAN implementations have considered different loss functions. Modifications to the full loss function have previously been proposed to improve machine learning model performance for image estimation. Examples include limiting the differences between the actual and estimated images through the 1-norm (22), SSIM (23), gradient difference (11), and image content (24). Additionally, Lin et al. proposed softmax cross entropy loss to prevent the mode collapse caused during training, and to reduce image blurriness in synthesised images (25). Here, we used the 1-norm (mean absolute error) and cross-entropy losses in our GAN implementation as our standard case and evaluated the addition of SSIM loss.

For the pix2pix model depicted in Figure 2, we used Binary Cross-Entropy for the adversarial loss and Mean Absolute Error (MAE), also known as 1-norm, for the pixel-wise loss. The total loss function (L_total_) is sum of the adversarial loss (L_adv_) and the MAE loss (L_MAE_) which are weighted by their respective hyperparameters (λ_adv and_ λ_MAE_):

$L_{\mathrm{total}}=\lambda_{adv}.L_{adv}+\lambda_{MAE}.L_{MAE}$

where $L_{adv}$ measures the difference between discriminator’s predictions between real and generated images according to:


$$L_{adv}=\frac{1}{N}\;\overset{N}{\underset{i=1}{\sum }}\left[y_{i}.\log\left(D\left(y_{i}\right)\right)+\left(1-y_{i}\right).\log\left(1-D\left(G\left(x_{i}\right)\right)\right)\right]$$

where $y_{i}$ is a ground truth label (1 for real and 0 for fake images), $D\left(y_{i}\right)$ is discriminator’s output for real images, $G\left(x_{i}\right)$ is a generated image corresponding to the input image $x_{i}$ , and finally $D\left(G\left(x_{i}\right)\right)$ is discriminator’s output for generated images. The MAE loss (L_MAE_) measures the absolute difference between the generated images and the corresponding ground truth images:


$$L_{MAE}=\frac{1}{N}\;\overset{N}{\underset{i=1}{\sum }}|G\left(x_{i}\right)-y_{i}|$$

where $G\left(x_{i}\right)$ is a generated image corresponding to the input image $x_{i}$ , $y_{i}$ is a ground truth image corresponding to $x_{i}$ , and N is a total number of images in the dataset.

### GAN training and testing

The dataset included a total of 48 patients from which 30 patient datasets were used for training, six for testing during training, and 12 datasets for validation which remained unseen to the model. The validation datasets were not used to train and test the model and included patient images with structural brain alterations following surgical resection. Here, one of our objectives was to establish how well the image synthesis algorithm, trained and tested using only structurally normal images, would perform in patients with abnormal brain structure. One hundred 2D slices were taken from each patient, resulting in a total of 4,800 2D slices for each imaging modality in this study. The GAN was trained using batch normalisation with a batch size of 8 and it was allowed to train for 1,000 epochs.

### Image quality metrics and statistical comparison

We employed RMSE, SSIM, and PSNR to quantify differences between the actual and estimated SPECT images as these are standard metrics used in cross-modality image synthesis studies (3). All metrics were computed for each synthesised unseen image slice and visualised using a violin plot across the entire test set of images. Statistical significance was set at *p* < 0.01 (denoted by *) and determined using a two-tailed, paired, t-test to evaluate the difference between the means of the two groups assessed.

## Results

**Figure 3:**
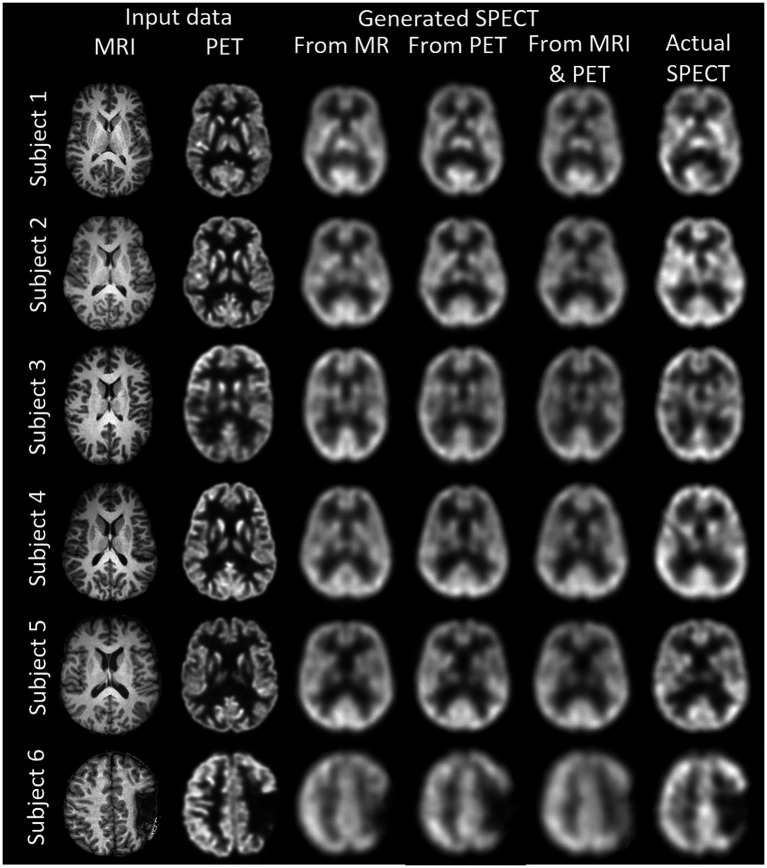
The native space image estimation result for select slices in five subjects using 1-norm and cross-entropy loss. Corresponding image metrics have been provided in Table 2. Subjects 1 to 5 are from the testing set, and Subject 6 is from the additional validation dataset.

**Figure 4:**
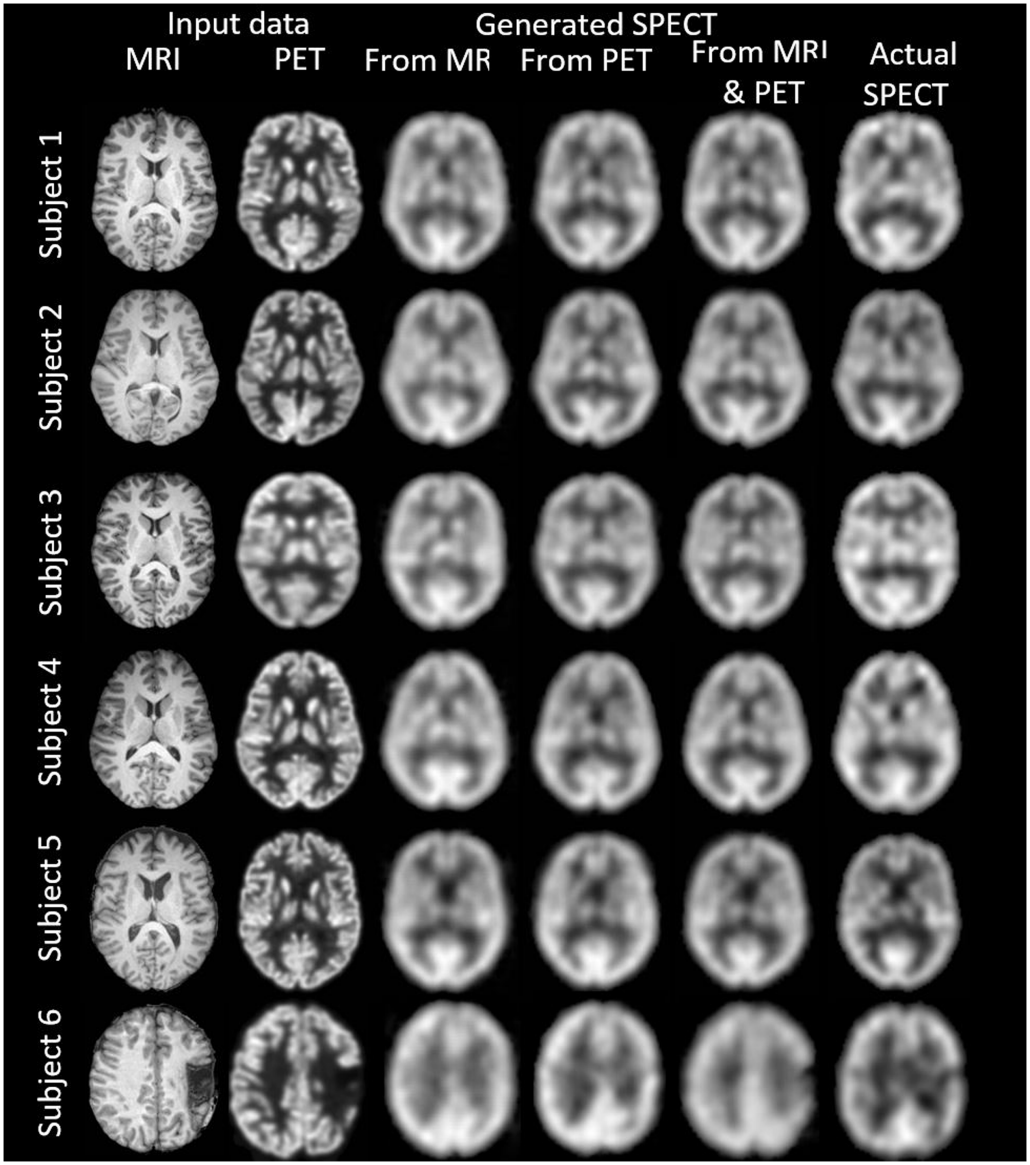
The template space image estimation result for select slices in five subjects using 1-norm and cross-entropy loss. Corresponding image metrics have been provided in Table 3. Subjects 1 to 5 are from the testing set, and Subject 6 is from the additional validation dataset.

We synthesised SPECT images from MRI, PET and using both MRI and PET as input to the GAN. Figures 3, 4 illustrate the native and template space image estimation results for example slices from five randomly chosen subjects. Image quality metrics are provided in Tables 2, 3, respectively. These results highlight that high quality SPECT images can be synthesised using MRI, PET or both image types as inputs. The mean and coefficient of variation for RMSE, SSIM, and PSNR for the 1,100 test image slices are listed in Table 4. While we describe further analyses of these results in specific contexts below, two key observations can already be made. First, native space input images tend to produce better synthesised images than images in template space. Second, the use of PET as input (either PET only or in combination with MRI) results in a lower coefficient of variation, suggesting that a PET input produces a more consistent SPECT image estimate than MRI alone. The differences between the results of using PET alone and PET combined with MRI as inputs are marginal and, based on these observations, it seems difficult to justify the use of a two-channel input (MRI and PET). Nonetheless, in what follows we provide the analysis for specific factors using MRI, PET and both MRI and PET as inputs to the GAN for SPECT image synthesis.

**Table 2:** The native space results for the three image metrics corresponding with the slices depicted in Figure 3.

	NATIVE SPACE																	
	MRI						PET						MRI & PET					
	Loss: 1-norm+cross-ent			Loss: +SSIM			Loss: 1-norm+cross-ent			Loss: +SSIM			Loss: 1-norm+cross-ent			Loss: +SSIM		
Subject	RMSE	SSIM	PSNR	RMSE	SSIM	PSNR	RMSE	SSIM	PSNR	RMSE	SSIM	PSNR	RMSE	SSIM	PSNR	RMSE	SSIM	PSNR
1	0.10	0.89	27.11	0.10	0.89	26.70	0.11	0.89	25.98	0.09	0.89	28.01	0.14	0.86	24.24	0.09	0.90	27.69
2	0.12	0.89	25.46	0.13	0.88	24.52	0.13	0.88	24.77	0.11	0.87	26.51	0.16	0.85	22.99	0.12	0.88	25.06
3	0.10	0.86	27.01	0.10	0.86	26.82	0.12	0.87	25.97	0.12	0.88	25.24	0.13	0.84	24.79	0.09	0.88	28.06
4	0.13	0.84	24.55	0.14	0.84	24.07	0.15	0.85	23.54	0.14	0.85	24.42	0.15	0.84	23.48	0.13	0.86	25.04
5	0.10	0.87	27.28	0.11	0.87	26.69	0.11	0.87	26.36	0.09	0.88	27.90	0.12	0.85	25.37	0.09	0.88	28.07
6	0.19	0.88	23.96	0.16	0.86	22.19	0.09	0.90	27.15	0.10	0.89	26.36	0.14	0.86	23.36	0.09	0.90	26.92

Results are shown when input images either MRI, PET, or both MRI and PET, with the standard loss function (1-norm plus cross-entropy) and when SSIM has been added (1-norm plus cross-entropy plus SSIM).

**Table 3:** The template space results for the three image metrics corresponding with the slices depicted in Figure 4.

	TEMPLATE SPACE																	
	MRI						PET						MRI & PET					
	Loss: 1-norm+cross-ent			Loss: +SSIM			Loss: 1-norm+cross-ent			Loss: +SSIM			Loss: 1-norm+cross-ent			Loss: +SSIM		
Subject	RMSE	SSIM	PSNR	RMSE	SSIM	PSNR	RMSE	SSIM	PSNR	RMSE	SSIM	PSNR	RMSE	SSIM	PSNR	RMSE	SSIM	PSNR
1	0.10	0.87	27.16	0.11	0.87	26.92	0.12	0.84	25.89	0.12	0.83	25.80	0.12	0.86	25.84	0.12	0.86	25.69
2	0.11	0.83	26.48	0.12	0.85	26.19	0.10	0.86	27.55	0.10	0.85	27.70	0.11	0.87	27.04	0.11	0.87	26.94
3	0.14	0.83	24.64	0.15	0.82	23.63	0.15	0.83	23.81	0.14	0.83	24.34	0.17	0.82	23.03	0.18	0.81	22.50
4	0.13	0.84	25.21	0.13	0.83	24.98	0.13	0.83	25.12	0.13	0.83	25.04	0.13	0.86	25.41	0.14	0.85	24.73
5	0.10	0.86	27.28	0.09	0.87	28.31	0.13	0.81	25.21	0.12	0.82	25.69	0.13	0.83	25.10	0.13	0.83	25.17
6	0.15	0.80	23.34	0.16	0.80	22.73	0.12	0.83	25.17	0.13	0.81	24.54	0.16	0.82	22.79	0.14	0.83	23.93

Results are shown when input images either MRI, PET, or both MRI and PET, with the standard loss function (1-norm plus cross-entropy) and when SSIM has been added (1-norm plus cross-entropy plus SSIM). Subjects 1 to 5 are from the testing set, and Subject 6 is from the additional validation dataset.

**Table 4:** Listed are the performance metrics when template and native space inputs are used, and without and with the addition of SSIM loss to the loss function, for synthesis of SPECT images from MRI, PET, and both.

Space / *Loss*	Metric	MRI	PET	MRI & PET
Template *1-norm + cross-ent*	RMSE	0.13 (38.46%)	0.12 (33.33%)	0.13 (30.77%)
	SSIM	0.82 (6.10%)	0.83 (6.02%)	0.83 (6.02%)
	PSNR	24.53 (11.01%)	25.12 (9.87%)	24.39 (8.69%)
Template *1-norm + cross-ent + SSIM*	RMSE	0.14 (35.71%)	0.12 (33.33%)	0.13 (30.77%)
	SSIM	0.83 (6.02%)	0.82 (6.10%)	0.83 (6.02%)
	PSNR	23.72(9.65%)	24.76 (9.13%)	24.27 (8.49%)
Native *1-norm + cross-ent*	RMSE	0.10 (30.00%)	0.10 (20.00%)	0.12 (16.67%)
	SSIM	0.87 (5.75%)	0.88 (3.41%)	0.86 (4.65%)
	PSNR	26.28 (9.74%)	25.92 (5.40%)	24.77 (5.85%)
Native *1-norm + cross-ent + SSIM*	RMSE	0.11 (27.27%)	0.09 (22.22%)	0.09 (22.22%)
	SSIM	0.86 (5.81%)	0.88 (3.41%)	0.88 (3.41%)
	PSNR	25.89 (9.15%)	26.99 (7.08%)	27.15 (6.85%)

For each metric the mean and the coefficient of variation (in apprentices) have been provided. Each value is based on 1,100 test image slices from 10 volumetric datasets not used for training and testing of the GAN. Subjects 1 to 5 are from the testing set, and Subject 6 is from the additional validation dataset.

### GAN training using native or template space

**Figure 5:**
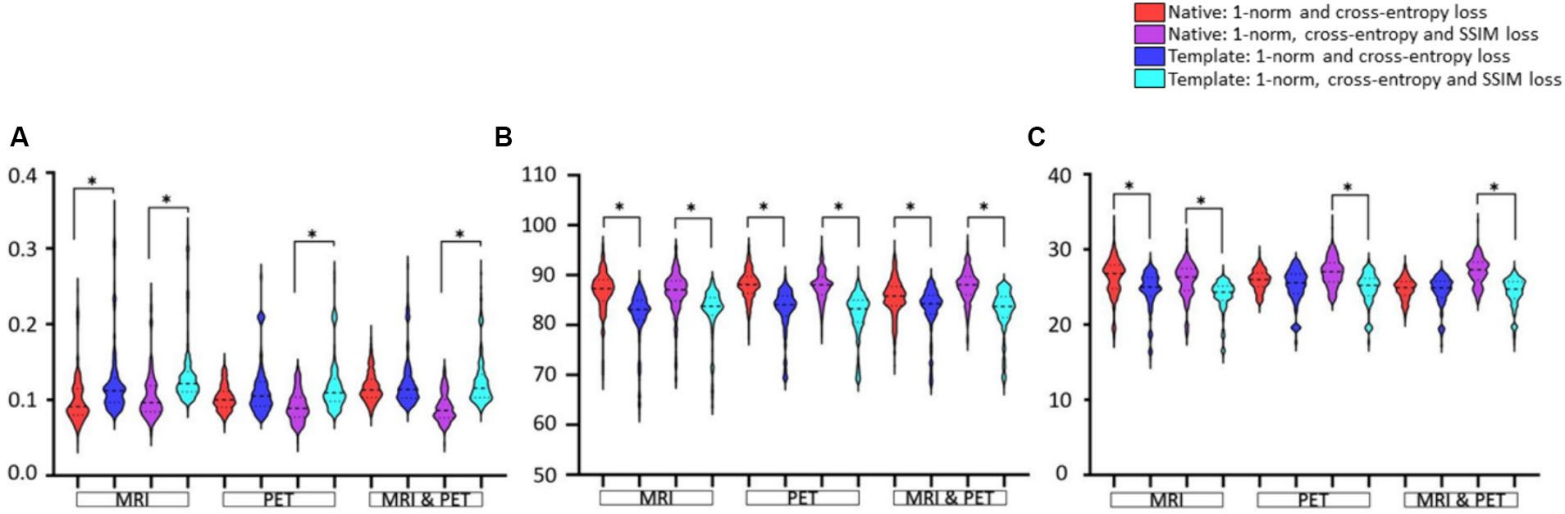
The native and template space results illustrated as violin plots and categorised by the input (x-axis). Shown are **(A)** RMSE, **(B)** SSIM as a percentage, and **(C)** PSNR. Both loss function implementations are provided. Here, *denotes *p* < 0.01 based on a student t-test.

In Figure 5, statistical comparisons of performance metrics between image synthesis using native or template space are provided. Significantly smaller RMSE, larger SSIM, and larger PSNR are seen for 14 of 18 comparisons. The RMSE using PET and MRI plus PET does not appear to differ significantly, see Figure 5A. There is also a tendency for native space input images to produce a larger PSNR than template space input images (see PET and MRI & PET in Figure 5C), however this was not statistically significant. Based on any of the image synthesis metrics evaluated, the use of native space input images never performed worse than the use of template space input images.

### Effect of additional SSIM loss function on image estimation

**Figure 6:**
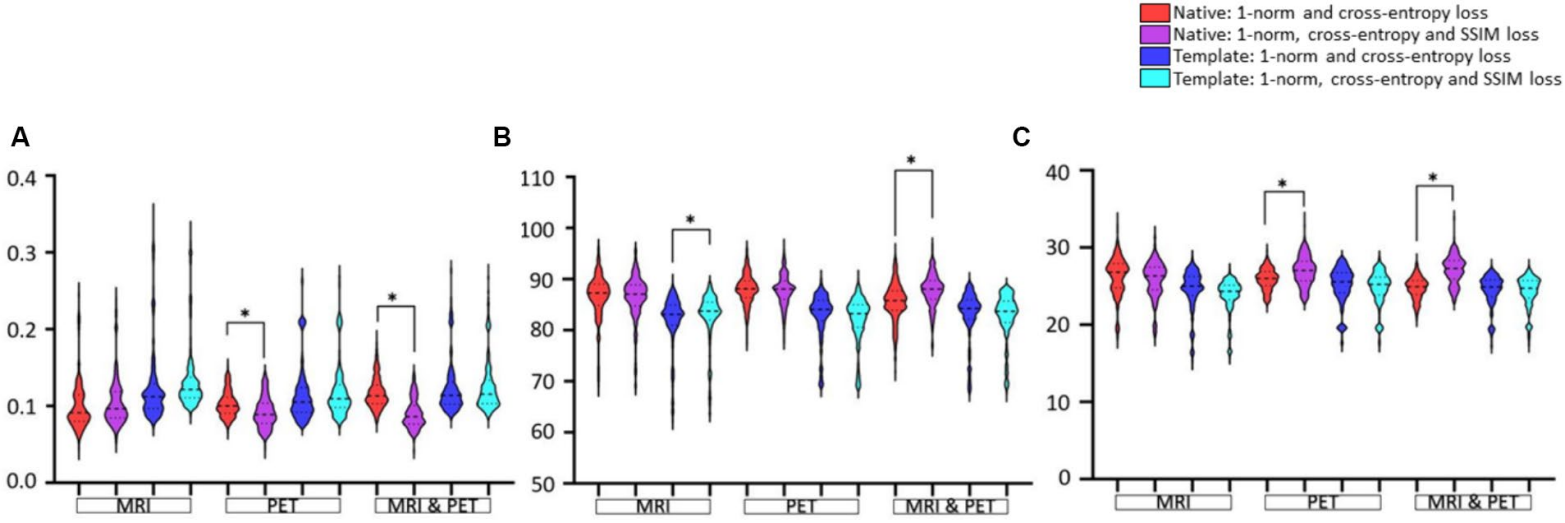
Evaluation of the choice of loss function used for GAN training. Results are illustrated as violin plots and organised in the categories of imaging modality, and adjacent violin plots of similar colour should be compared. Shown are image metric results for **(A)** RMSE, **(B)** SSIM, and **(C)** PSNR. Here, *denotes *p* < 0.01 based on a two-tailed paired t-test comparison of means and the dashed line is the median with the dotted line identifying the interquartile range.

Data presentation in Figure 6 aims to facilitate the comparison between loss functions based on RMSE, SSIM and PSNR image metrics. The only notable impact of adding SSIM loss is on estimation based on input PET (RMSE and PSNR improve significantly) or MRI and PET (SSIM and PSNR improve) images in native space. Otherwise, adding SSIM loss to 1-norm and cross-entropy losses does not appear to benefit GAN training.

### Choice of image modality as input for SPECT synthesis

**Figure 7:**
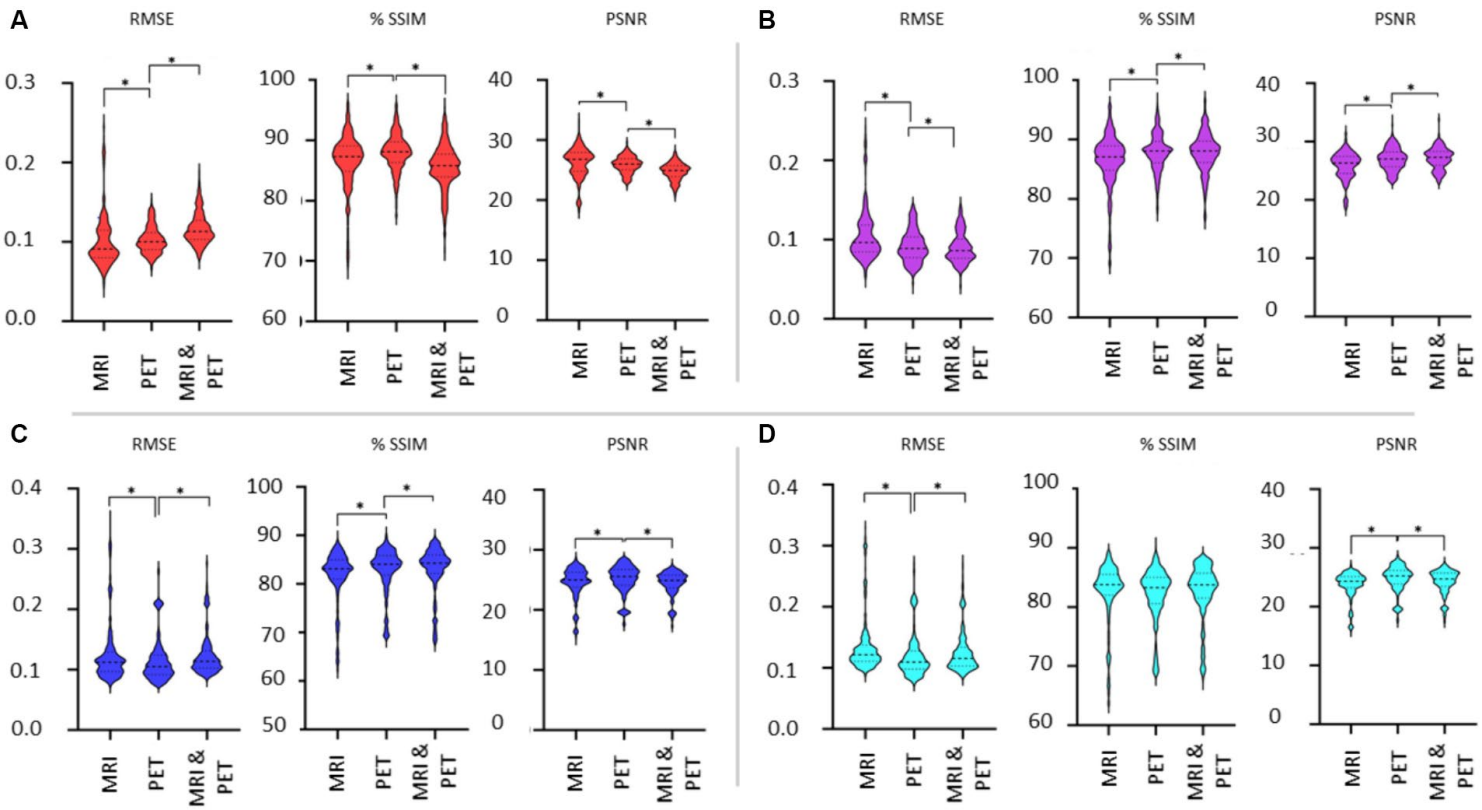
Evaluation of the choice of imaging modality used as input to the GAN. Results are illustrated as violin plots and organised by the imaging modality input (x-axis). Shown are results for **(A)** native space with 1-norm and cross-entropy loss, **(B)** native space with 1-norm, cross-entropy and SSIM loss, **(C)** template space with 1-norm and cross-entropy loss, and **(D)** template space with 1-norm, cross-entropy and SSIM loss. Here, *denotes *p* < 0.01 based on a two-tailed paired t-test comparison of means and the dashed line is the median with the dotted line identifying the interquartile range.

Figure 7 shows the violin plots for the four cases considered: input images in native and template space without and with the addition of SSIM loss. Comparisons are based on the imaging modality used to train the GAN. We expect both SSIM and PSNR to decrease (i.e., image quality worsens) as RMSE increases. From Figure 6 we concluded that the addition of SSIM loss does not improve image estimation quality. However, in Figure 7 the case with SSIM loss makes the predictions more consistent across the various imaging modalities.

The use of template instead of native space images diminishes the differences in the metrics when considering the different imaging modalities as input into the GAN. We should note that while statistically significant differences in the mean of the RMSE, SSIM and PSNR metrics have been identified between MRI, PET and dual input cases, it is difficult to discern potentially impactful image quality differences between whether MRI or PET is used for image synthesis of SPECT (refer back to Figure 4). Benefits of using MRI and PET together as a dual input into the GAN are lacking based on Figure 7. Notably, the best image synthesis SPECT result is achieved when native MRI is used as the input into the GAN, which is significantly better than using PET or both MRI and PET (see RMSE and PSNR in Figure 7A). However, based on the results presented, one could be indifferent between choosing MRI and PET.

## Discussion

We performed the synthesis of intra-ictal SPECT as this scan is necessary for clinical management of epilepsy patients, comes with additional exposure to radiation, and can be an unreliable baseline measure because the time since last seizure may not be available. We implemented a machine learning framework to synthesise SPECT from PET and/or MRI images using a GAN. Our results confirm that interictal SPECT can be generated using either modality alone or in combination. MRI and native space images with 1-norm and cross-entropy loss in the GAN framework led to the best image synthesis results. Interestingly, SPECT could be synthesised from PET almost equally well. An additional important finding is that native space input training images produce superior results than template space training images. This result implies that affine image registration between subjects and between imaging modalities is not necessary. Our detailed analysis did not suggest that multi-modality input (i.e., MRI and PET together) improved SPECT image synthesis.

### Can MRI and PET synthesise high quality SPECT?

Our results reaffirm (3) that a properly chosen GAN can be used effectively in cross-modality image synthesis. The impact of registering images to template space versus using broadly aligned native space images has not been evaluated previously. We found SPECT synthesis based on native space images was significantly superior to that using template space input images. Previous studies have not synthesised SPECT from either MRI or PET, but synthesis of PET from MRI has been performed. The studies reported PSNR of 29.33 (20) and 24.49 (19), which is consistent with our PSNRs of 26.28 and 27.15 for the synthesis of SPECT from MRI and PET.

Our method will likely work with other types of MRI sequences, including T2-weighted MRI, as this problem still involves a similar image synthesis scenario, but the GAN would have to be retrained to learn the new cross-modality image intensity projection. A remaining question is whether replacing T1-weighted with T2-weighted MRI or employing both MRI sequences as a two-channel input can benefit the quality of the image estimation results achieved here. Previously Wei et al. suggested that estimation of PET using multi-sequence MRI input images resulted in the fusion of complex information, yielding improved results over single MRI sequence inputs (17). In Chen’s study (26), however, prediction of high dose PET using low dose PET and multiple MRI sequences (i.e., T1-weighted, T2-weighted & T2 fluid attenuated inversion recovery) yielded similar results to prediction using only T1-weighted MRI by DaCosta-Luis and Reader (27). Based on these mixed findings, multi-sequence MRI should be evaluated separately for different image estimation tasks and datasets. In our study, estimation of the SPECT image from T1-weighted MRI and PET together (i.e., multi-channel input to the GAN) had slightly superior performance, only in the native space implementation. The use of T2-weighted MRI images as input to the GAN may provide similar results but should empirically be tested in the future. It appears that both single sequence MRI and PET have sufficient structural information for the GAN to adequately learn the SPECT image synthesis mapping.

### Choice of image space for image synthesis

While most studies have registered subject specific images to a template space (19, 20, 28), only a few have applied methods in native space (10, 29). To our knowledge, the choice of using template space versus native space images as input to the GAN has not been evaluated, nor has a justification been provided for the choices made in the disparate studies. For this reason, we evaluated the two approaches. Our findings conclude that image estimation in native space performs better than in template space. During training and testing, the initial epochs had a better loss function convergence in template space, but with increasing epochs the native space loss value tended to a lower loss value. This observation may indicate learning using native space images is a more diverse problem, the result of which is broadening of image projections leading to higher accuracy.

### Clinical relevance of the findings

In pre-surgical evaluations of epilepsy, precise localisation of epileptogenic zone is critical for achieving a favourable surgical result and to reduce the surgery side effects (30). Interictal SPECT is typically acquired following patient discharge from hospital where they are not monitored by medical staff or video-EEG. Therefore, confirmation of seizure events within that period is doubtful. Any unknown seizure event or seizure propagation in the past 24 h prior to radiotracer injection of interictal SPECT can impact localisation of the epileptogenic zone (31). Our cross-modality image synthesis approach to generate interictal SPECT from MRI or PET may become a better reference image (i.e., synthesised interictal SPECT) preventing ambiguity regarding the accuracy of a normal brain activity. This approach may improve the clinical workflow by removing the interictal SPECT scan, reduce nuclear medicine workloads, reduce the cost to the patient and to the healthcare system, and remove exposure to radiation associated with the interictal SPECT scan. The next step for future clinical evaluation would be to process SISCOM (subtraction of ictal SPECT from interictal SPECT, co-registered to MRI) using both real and synthetic interictal SPECT images and to compare the reliability with which nuclear medicine physicians localise the seizure onset zone using both types of images.

### Considerations

Careful review of previous studies highlights the fact that only increasing the number of training datasets in the machine learning model does not result in better image synthesis quality (17, 19, 28). A lack of data for training and testing of the GAN is unlikely to be a limitation of this study, as the large number of test and training image slices provides adequate variability for robust learning. Improving image synthesis performance is more likely dependant on other factors including the machine learning model architecture, choice of loss functions such as 1- and 2-norms to minimise noise and blurriness, setting an appropriate learning rate to optimise the learning process, choosing an optimal batch size to improve the learning quality and to be compatible with computational resources (17). We modified the learning rate, batch size (32), and adopted L1-norm loss to avoid blurriness (22). Furthermore, we evaluated the addition of SSIM loss, which based on our findings, did not benefit SPECT image synthesis.

We synthesised 2D images instead of 3D volumes. A motivation for this approach was to create a larger number of training and testing pairs. Stacking of synthesised 2D slices to form 3D volumetric images can lead to through-slice signal variations, which was not considered here. However, image intensity variations across slices can be corrected using intensity normalisation, noise filtering, and inhomogeneity field correction (33).

## Conclusion

The cross-modality image synthesis of SPECT images using a GAN has not been considered before. We implemented a GAN to synthesise SPECT images from MRI, PET, and using both MRI and PET as a dual channel input. We performed experiments in native and template spaces and added SSIM loss to the 1-norm and cross-entropy loss function. We found that high quality SPECT images can be synthesised from MRI and PET, and using both imaging modalities as input into the GAN. The best results were generated using MRI in native space as the input without the addition of SSIM loss to the GAN framework. Notably, the use of PET images instead of MRI performed almost equally well for the SPECT synthesis task. Our interesting results suggest that registration of subject specific images to a template space does not increase image synthesis performance, while improving the machine learning workflow in cross-modality image synthesis. Our image synthesis of SPECT from MRI and/or PET may find use in epilepsy management where the baseline intra-ictal SPECT is unreliable or not available.

## Data availability statement

The datasets presented in this article are not readily available because of ethical and privacy restrictions. Requests to access the datasets should be directed to the corresponding author.

## Ethics statement

The studies involving humans were approved by the Human Research Ethics Committee of the Royal Brisbane & Women’s Hospital. The studies were conducted in accordance with the local legislation and institutional requirements. The ethics committee/institutional review board waived the requirement of written informed consent for participation from the participants or the participants' legal guardians/next of kin due to the retrospective nature of the study.

## Author contributions

AF: Conceptualization, Data curation, Formal analysis, Funding acquisition, Investigation, Methodology, Project administration, Resources, Software, Supervision, Validation, Visualization, Writing – original draft, Writing – review & editing. DR: Conceptualization, Data curation, Formal analysis, Funding acquisition, Investigation, Methodology, Project administration, Resources, Software, Supervision, Validation, Visualization, Writing – original draft, Writing – review & editing. SR: Conceptualization, Data curation, Formal analysis, Funding acquisition, Investigation, Methodology, Project administration, Resources, Software, Supervision, Validation, Visualization, Writing – original draft, Writing – review & editing. StG: Data curation, Project administration, Resources, Writing – review & editing. SoG: Data curation, Methodology, Writing – review & editing. VV: Supervision, Validation, Visualization, Writing – original draft, Writing – review & editing, Conceptualization, Data curation, Formal analysis, Funding acquisition, Investigation, Methodology, Project administration, Resources, Software.
